# Integrating Experiment and Theory to Understand TCR-pMHC Dynamics

**DOI:** 10.3389/fimmu.2018.02898

**Published:** 2018-12-07

**Authors:** Ashley M. Buckle, Natalie A. Borg

**Affiliations:** Infection and Immunity Program, Monash Biomedicine Discovery Institute and Department of Biochemistry and Molecular Biology, Monash University, Clayton, VIC, Australia

**Keywords:** TCR, MHC, conformational dynamics, T cell activation, immune synapse, TCR recognition

## Abstract

The conformational dynamism of proteins is well established. Rather than having a single structure, proteins are more accurately described as a conformational ensemble that exists across a rugged energy landscape, where different conformational sub-states interconvert. The interaction between αβ T cell receptors (TCR) and cognate peptide-MHC (pMHC) is no exception, and is a dynamic process that involves substantial conformational change. This review focuses on technological advances that have begun to establish the role of conformational dynamics and dynamic allostery in TCR recognition of the pMHC and the early stages of signaling. We discuss how the marriage of molecular dynamics (MD) simulations with experimental techniques provides us with new ways to dissect and interpret the process of TCR ligation. Notably, application of simulation techniques lags behind other fields, but is predicted to make substantial contributions. Finally, we highlight integrated approaches that are being used to shed light on some of the key outstanding questions in the early events leading to TCR signaling.

## Introduction

**Figure 1:**
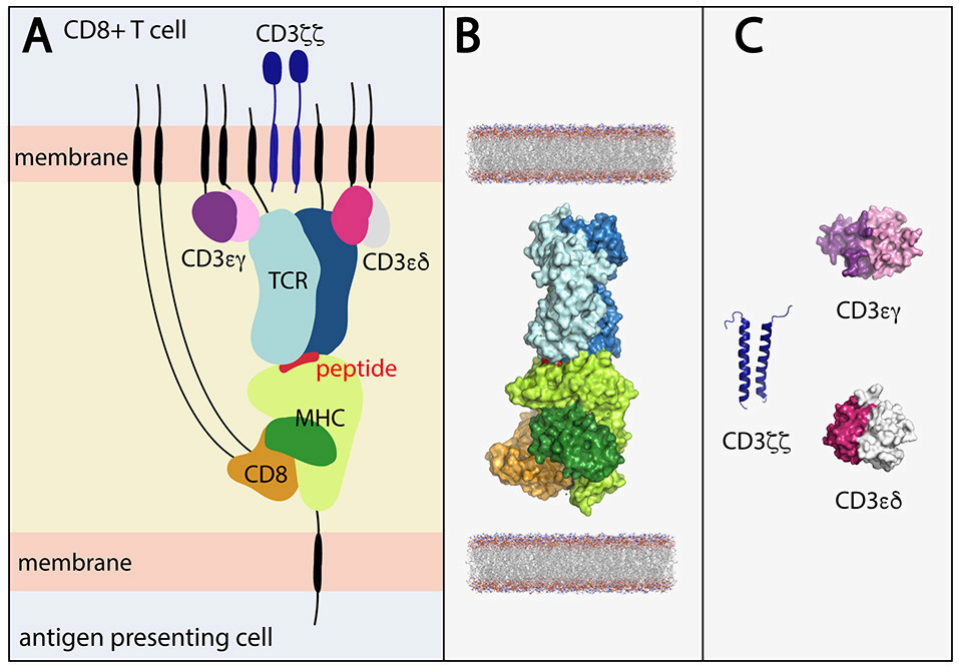
**(A)** Stylized view of the pMHC-TCR and core components required for T cell signaling. **(B)** Components of the pMHC-TCR complex for which structures have been determined in combination with one another. The complex depicts peptide-bound HLA-A*02 in complex with CD8 and the B7 TCR (derived by superimposing components of PDB ID's 1AKJ and 1BD2. **(C)** Portions of the CD3εγ (ectodomain; PDB ID 1SY6), CD3εδ (ectodomain; PDB 1XIW) and CD3ζζ (TM domains; PDB ID 2MAC) signaling components have been structurally determined, but not in complex with the TCR. Black lines represent regions of conformational flexibility. TCR α and β chain shown in dark and light blue, respectively. MHC class I heavy and light (β_2_-microglobulin) chain shown in light and dark green, respectively. Peptide shown in red. CD8αα shown in orange.

Self or foreign intracellular peptides are presented on the surface of antigen presenting cells (APC) by major histocompatibility complex (MHC) class I molecules. These peptide-bound MHC (pMHC) molecules undergo surveillance by CD8+ cytotoxic T lymphocytes (CTLs) via the αβ T cell receptors (TCR) that are expressed on their surface. TCR engagement of the pMHC leads to the formation of an immune synapse that is central to T cell activation (Figure 1A). The outcome of T cell engagement with the pMHC influences T cell fate, playing a role in the defense against infection and cancer, but on the flip side, allergic reactions, autoimmune disease, transplant rejection, and drug hypersensitivity. Despite the importance of T cell activation, we have a poor understanding of how the TCR-pMHC initiates an intracellular signal and this impedes our ability to manipulate the T cell response to target infection and cancer. What is clear however is that there is enormous complexity to the overall response and dissecting it requires the integration of a diverse suite of both experimental and computational tools and techniques.

Due to the relatively small size of the TCR-pMHC, X-ray crystallography has led the way in the structural determination of the extracellular domains of pMHC and TCR alone or in complex with one another at near-atomic resolution. These studies detail the conformation of the peptide, its interactions with MHC as well as the TCR and structural changes the MHC and/or peptide undergoes upon TCR binding. It was long-anticipated that these accrued X-ray structures would also reveal how the information at the pMHC interface is communicated from the variable domains to the membrane proximal constant domains and via the CD3 subunits necessary for signal transduction. A clear mechanism however has not been revealed, exemplified by instances where single amino acid changes in a peptide produce near-identical structural snapshots but different T cell outcomes (1–3). This indicates additional factors, concealed by X-ray crystallographic snapshots, are at play.

**Figure 2:**
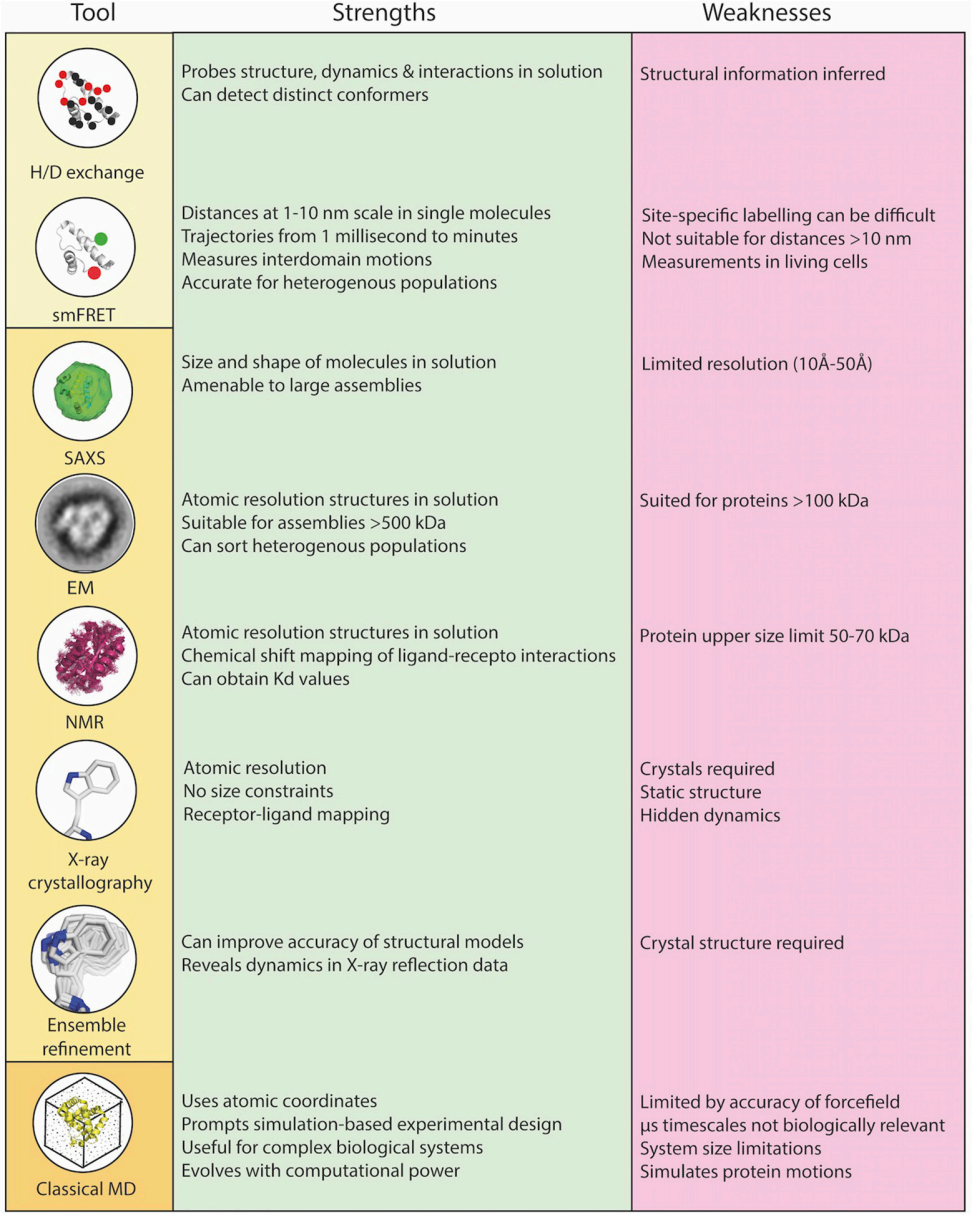
Combinations of biophysical, structural, and computational techniques are a necessity to overcome the limitations of each individual technique and to rigorously understand the role of dynamics in TCR-pMHC function at the core of the immunological synapse. Biophysical techniques (yellow box), structural techniques (light orange box), computational techniques (dark orange box).

There is emerging evidence that conformational dynamics and dynamic allostery influences T cell recognition and activation (4–6), yet until recently, this has been overlooked in our effort to understand the structural basis of TCR recognition of the pMHC. The importance of conformational dynamics at the immune synapse has been the subject of excellent recent reviews [see, for example (7–12)]. In this review, we instead focus on relevant methodologies (highlighted in Figure 2), and specifically recent advances in computational, structural and biophysical techniques, and how they can be integrated to provide powerful insights into the key early stages of the TCR-pMHC interaction. Finally, we highlight integrated approaches that are being used to shed light on some of the key outstanding questions in the early events leading to TCR signaling.

## X-ray Crystallography: Pioneering Atomic Resolution Details

Typically, the structural flexibility of a TCR-pMHC system is solely interpreted from a single set of coordinates derived from X-ray diffraction data. In this case flexibility is merely inferred mainly by comparing structural differences between TCR-pMHC and their unbound constituents (13, 14) and the consideration of atomic temperature (B) factors. B-factors can be used to estimate atomic displacements that arise from static and dynamic disorder (alternative conformations in the crystal lattice, and atomic fluctuations in the crystal, respectively). By comparing identical molecules in different crystal lattices, the influence of crystal packing on protein structure can be analyzed (15–17). Indeed, crystal packing can select radically different conformations from a heterogeneous ensemble, giving clues to conformational dynamics (16). For example, structural variation of HLA-B^*^35:08-LPEP with SB27 TCR within two crystal forms suggested a “scanning” motion of the TCR on pMHC that was further supported and extended by molecular dynamics (MD) studies (3, 18).

Due to crystal packing and data collection at cryogenic temperatures, B-factors underrepresent the amplitude of conformational populations (19). Thus, due to the failure of current refinement algorithms to model structural heterogeneity, the analysis of single, static crystallographic models can reveal limited information on the dynamics of the system in solution (20–22). However, there are examples where flexibility insight has been successfully obtained and useful correlations made. For example, B-factor analysis of structures of HLA-B^*^35:01 and HLA-B^*^35:08, that differ by a single amino acid, but are bound to the same Epstein-Barr virus (EBV) peptide (HPVG) provided insights into the influence of MHC polymorphism on peptide mobility and the T cell response (23). Likewise, the structural comparison of a TCR in its unbound vs. pMHC-bound state revealed a conformational change in the A-B loop of the Cα domain that borders the CD3ε binding site (24) that was later verified to correlate with pMHC ligation (25).

In addition to concealing flexibility, the X-ray structures available are also incomplete and lack the core components necessary for signal transduction (Figures 1B,C). While the structures of TCR-pMHC, pMHC-CD8 (26–29), CD3 heterodimers (30–34), and CD8 homo/heterodimers (35, 36) have been determined, critically informative complexes such as CD8 and/or CD3 in complex with TCR-pMHC or even just TCR-CD3 are lacking due to the poor affinity of soluble CD3 and CD8 for the TCR and MHC, respectively (33, 37, 38). Also absent, due to technical challenges, are the stalk regions, membrane-spanning domains, and intracellular tails of the TCR, MHC, and CD8 and CD3 molecules, which play a role in complex assembly, the spatial organization of the components and signal transmission (39–46). Therefore, whilst X-ray crystallography can yield highly informative, high-resolution structures, its limitations necessitate the use of clever engineering and complementary techniques to make the next leap in terms of T cell signaling.

## Molecular Dynamics Simulations: Producing Testable Hypotheses and Placing Experimental Findings into a Theoretical Framework

Although conventional X-ray crystallographic analysis provides little information regarding dynamics, the exquisite resolution as well as model completeness has provided a solid data foundation that has spurned an increasing amount of MD simulation studies. All-atom MD simulations probe the flexibility of the system by computing iterative solutions of Newton's equations of motion over time (47). The raw output of MD simulations—trajectories, describe the atomic positions at time-points during the simulation. Unfortunately however, MD is computationally demanding and limits the technique to examination of relatively short time spans (e.g., a microsecond), orders of magnitude shorter than more biologically relevant timescales over which many larger motions occur. The signal, for example, produced following T cell recognition of a pMHC is of the seconds to minutes timescale (48–52). Several approaches have emerged that allow this limitation to be mitigated somewhat and here we discuss briefly below the most popular and useful approaches. Replica exchange MD (REMD) can improve the sampling by simulating multiple copies of the same molecule at different temperatures, allowing an unbiased way of improving conformational sampling (53). REMD is computationally intensive and therefore currently limited to relatively small systems, for example to investigate how polymorphic amino acid differences between allotypes alter the conformational plasticity of the MHC class I binding pocket (54). Alternatively, steered MD (SMD) applies an external force to the protein to study its mechanical response, analogous to atomic force microscopy (55). This method is well suited to studying protein-protein interactions, and has been used to investigate TCR-pMHC dissociation (56–58). A novel use of SMD simulations based on agonist and non-agonist complex crystal structures was to develop a molecular model of TCR-pMHC “catch bond” formation (12). Catch bonds represent a net accumulation of molecular interactions under force, revealing an additional level of dynamic diversity built-in as a proofreading mechanism to link TCR recognition and subsequent activation. The related approaches of targeted MD (TMD) and umbrella sampling apply forces in order to promote new conformations, and are used to predict a pathway between two known conformations. Such “pulling” simulations, although not used to study dynamics *per se*, have been used to estimate the binding free energies between peptides and MHC (59). The atomic complexity of the system can be reduced significantly using coarse-grained (CG) methods in which groups of atoms are replaced by beads, allowing longer simulations at the cost of fine details (60). This approach is therefore useful for studying larger complexes such as TCR-pMHC in membrane (61) and TCR-pMHC-CD4 complexes (62). CG methods are complimentary to atomistic simulations and offer a feasible approach to tackling the combined challenges of large immune assemblies and long timescales associated with changes in membrane morphology. Accuracy of MD simulations are dependent upon available force fields—mathematical-physical descriptions of a system used to calculate the forces acting upon all atoms in order to solve Newton's equations of motion. Force fields, though improving constantly, have known imperfections (63–65), so currently it is preferable to seek experimental validation. Getting stuck in local energy minima is a particular limitation, but energy landscapes can be sampled more efficiently using advanced adaptive sampling techniques such as Markov state models (66), allowing the identification of metastable states.

In summary, MD simulation is an increasingly important member of the toolbox, since it is particularly well suited to producing experimentally testable hypotheses as well as placing existing experimental findings into a theoretical framework. Despite the advances in enhanced sampling methods and possible simulation of larger complexes, MD simulations of experimentally-determined TCR-pMHC structures have not been widely adopted.

## Ensemble Refinement: Using MD Simulations to Extract Dynamics from the Crystalline State

Another way to explore protein dynamics from experimental diffraction data is by simultaneously performing short molecular dynamics simulations with structure refinement (20, 67, 68). This method, known as ensemble refinement, produces an ensemble of models of the same structure that provides extended biological insight, often whilst improving the refinement statistics [i.e., free R-factor (*R*_free_)]. Although the ensemble refinement method has long been established and is well-validated (69–72), it is not routinely incorporated as a tool to analyze the X-ray structures of pMHC class I systems. This prompted our re-analysis of 11 published systems to reveal the dynamics present in the X-ray data, revealing the benefits of incorporating ensemble refinement to the structural interpretation (73).

**Figure 3:**
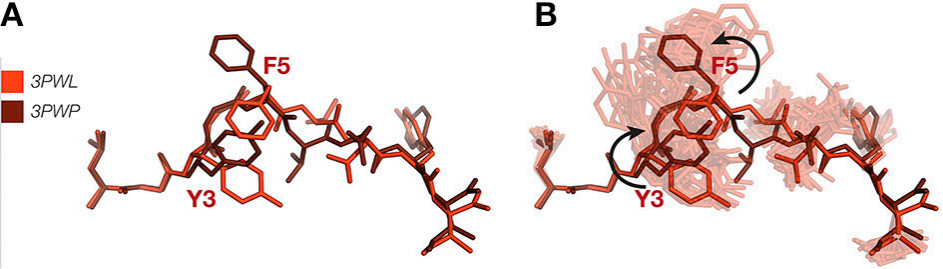
**(A)** Superposition of HuD-HLA peptide (red) (PDB ID 3PWL) and TCR-HuD-HLA peptide (brown) (PDB ID 3PWP) showing putative TCR binding-induced bond rotations. **(B)** Ensemble refinement show both these variants occur in the pMHC ensemble. MHC α-helices have been omitted.

A pertinent example of how ensemble refinement can extend and enrich existing crystallographic interpretations relates to the induced fit vs. conformational selection model of TCR binding to pMHC. In the induced fit model, the TCR undergoes a conformational change upon binding the pMHC, whereas in the conformational selection model a conformation that is compatible with binding is selected from an ensemble of conformations (74). Borbulevych et al. (75) sought to understand the causes of the cross-recognition of self- and non-self-peptides by the A6 TCR, by comparing the structures of the self-peptide HuD and the non-self-peptide Tax, both when bound to MHC HLA-A^*^02 alone and in a MHC-TCR complex. While the bound conformations are very similar for both peptides, differences in the orientation of the p3 and p5 side chains necessitate that the HuD peptide must undergo a conformational change in order to bind to the TCR (Figure 3A), while the Tax peptide does not. Our ensemble results, however, show that the Tyr3 and Phe5 residues in the HuD peptide are flexible enough to convert between the two conformations (Figure 3B). This indicates that the differences found between the MHC and TCR-pMHC conformations may be due to intrinsic flexibility rather than any change elicited by binding itself, and suggests that differences between static pMHC and TCR-pMHC may be due to a combination of the inherent flexibilities of each system, and the complexation process. Static structures may bias the interpretation in favor of an induced fit mechanism, whereas analysis as a conformational ensemble can allow also for conformational selection. Furthermore, reliance on single crystallographic structures of pMHC, with or without TCR, entails pitfalls for understanding the rules of productive TCR ligation, particularly for static interpretations involving fine details such as interaction networks and side chain orientation. Since in the worst cases, it may fail to properly distinguish real results from noise, it supports a view that biological observations should be explained through the properties of ensembles rather than isolated structures, as these are less prone to observer bias.

While we tend to describe TCR binding to the pMHC as either undergoing induced fit or conformational selection, the likelihood is that the TCR binds through a combination of both models. Scott et al. (6), highlight using time resolved fluorescence measurements, MD and structural and thermodynamic data that the CDR3 loops of a TCR can have varying degrees of flexibility. In the case of the A6 TCR the CDR3β loop is highly flexible and can rapidly sample ligands with a range of conformations, whereas the CDR3α has a slower motion that restricts the repertoire of peptides that it can bind. Therefore, the two models are not necessarily mutually exclusive, but instead describe a continuum (74).

Computing requirements of ensemble refinement are typically much greater than for single structure analysis, likely explaining the relatively slow adoption of this technique. However, continual improvements in refinement software [notably Phenix, which allows straightforward and user-friendly ensemble refinement from a PC (76)] and hardware have now placed this technique easily within the grasp of most structural immunologists. For example, in a recent ensemble refinement analysis of pMHC structures, refinement could be completed in < 3 h for most systems, using a typical off-the-shelf desktop computer (73).

## Nuclear Magnetic Resonance Spectroscopy (NMR): Atomistic Dynamics in Solution

NMR measures the absorbance and re-emission of electromagnetic radiation by nuclei in a magnetic field, and has provided significant information on protein structure and dynamics for the past 30 years. The use of NMR spectroscopy to study pMHC dynamics is complicated by the relatively large size of these systems (77), however several recent studies that have characterized TCR-pMHC binding have all found that significant conformational variation exists in the TCR, peptide, and MHC (78). In addition to noting conformational changes at the TCR-pMHC interface, conformational variation was also observed at remote sites, including within the membrane-proximal Cβ domain of the TCR, that implies an allosteric mechanism in TCR signaling (79), and the β_2_m (β_2_-microglobulin) binding site on the MHC that was sensitive to MHC polymorphism and the bound peptide (80). Notably, each of these NMR studies benefitted from mapping chemical shift perturbations onto available X-ray structures, but revealed flexible regions not otherwise observed from the X-ray structures alone. NMR has also been used to validate MD predictions that show long-range allosteric communication between the TCR binding sites for pMHC and CD3, a key step in early T-cell activation (81). These NMR studies reiterate the need to characterize the TCR-pMHC system as an allosteric ensemble in which ligand binding alters the energy landscape of the entire ensemble. In an allosteric ensemble conformational changes that concurrently occur at distal sites, but do not necessarily dominate the ensemble, can be mapped to reveal cooperativity between sites, or dynamically-driven allostery, revealing previously hidden and unforseen insights into signal transmission (82–84).

## Fluorescence Spectroscopy: Probing Environmental Dynamics and Distances

The intrinsic fluorescence of aromatic (usually tryptophan) residues is sensitive to their environment, and can therefore be used to monitor dynamics. Fluorescence anisotropy, in particular, has become a powerful method with which to study pMHC-TCR dynamics, especially when coupled with other techniques (85–87). Dynamic insight has also been gained by Förster resonance energy transfer (FRET), in which energy transfered between a donor and an acceptor chromophore is used to measure distances between chromophore-labeled residues as a function of time, particularly powerful when combined with structural techniques (88–90). An elegant example of integrating experimental and computational approaches, from the protein folding field, combined small molecule (sm) FRET with advanced MD simulations and machine learning (91). Such a combined approach has not yet been reported for pMHC-TCR systems, but clearly holds much promise. Nevertheless, there are other examples pertaining to the value of the use of fluorescence to study pMHC-TCR systems. For example, site-directed fluorescence labeling, in which an extrinsic fluorescent probe is attached to a cysteine residue, has been used to note that the A-B loop within the TCR Cα domain undergoes a conformational change upon pMHC ligation (25). FRET has also been used to measure intermolecular associations in live cells. Yachi et al. (92) measured the molecular interaction between TCR-CD3ζ and CD8 on antigen presenting cells loaded with different peptides, revealing structurally similar peptides alter the kinetics of the CD8-TCR interaction and translate into differential T cell responses. Another study used a FRET sensor to map the spatiotemporal dynamics of protein clustering in live T cells, linking the molecular density of TCR clusters with TCR triggering (93). Clearly, our understanding of TCR-pMHC systems could benefit from the further integrated use of intramolecular and intermolecular FRET sensors, particularly when coupled with structural data.

## Hydrogen/Deuterium (H/D) Exchange: Solvent Accessibility and Local Dynamics

H/D exchange involves a steady-state reaction in which deuterium atoms replace covalently bonded hydrogen atoms that are not participating in H-bonds. The rate of that exchange is usually measured by mass spectrometry, providing information on solvent accessibility and local dynamics. A study combining H/D exchange, fluorescence anisotropy, and structural analyses, showed that the flexibility of the peptide binding groove of the class I MHC protein HLA-A^*^02:01 varies significantly with different peptides (85). Further evidence for the role of conformational plasticity in peptide selection by MHC Class I was revealed by comparing H/D exchange of two allotypes in peptide-bound and free states (94). H/D exchange has also been used to probe the dynamics at the TCR-pMHC interface, with several studies highlighting that conformational flexibility is contingent upon the MHC allele (95), the bound peptide (85), and upon TCR ligation (96) and all of which have implications for T cell signaling.

## Small Angle X-ray Scattering (SAXS): Low Resolution Structure in Solution

Despite inherent limitations in resolution that can be achieved, SAXS can be used to study the size, shape and assembly of proteins, without the size limitations of other techniques such as NMR (97). In a monodisperse solution, geometric parameters such as the maximum particle dimension (*D*_*max*_), radius of gyration (*R*_g_), and the forward scattering intensity, *I(0)*, can be calculated from SAXS data; these values can serve as a point of comparison with the dimensions provided by a crystal structure or when studying the same protein under various experimental conditions or in a liganded vs. unliganded state. For example, in conjunction with other techniques and by comparing the *R*_g_ of HLA-DR1 (MHC class II) bound to a wild-type peptide, or a weak- or tight-binding peptide variant Yin et al. (98, 99) correlated pMHC conformational differences with susceptibility to peptide exchange by the non-classical MHC class II molecule HLA-DM. In another pMHC class II system, SAXS was used to show the pathogen-derived proteins, Salp15 and gp120, caused binding-induced conformational changes in CD4 that subsequently influence CD4+ T cell activation during infection (100, 101).

SAXS can also be used to characterize polydisperse systems such as modular proteins with flexible linkers or proteins bearing disordered regions. The ensemble optimization method (EOM) (102) is one approach to describe this experimental SAXS data. It generates a pool of *n* independent models based upon the sequence and structural information of the target and then selects a subset of ensembles that best describe the experimental SAXS data. The distributions of the properties of the selected ensembles, including *R*_g_ (radius of gyration), *D*_*max*_ (maximum particle dimension), *R*_*flex*_ (measure of flexibility) and *R*_σ_ (variance of the ensemble distribution with respect to the original pool), can then be compared to those of the pool of independent models to assess the flexibility of the system. To the best of our knowledge EOM has not yet been used to study TCR-pMHC systems, despite both MHCs and TCRs being multidomain proteins with flexible linkers. It is thus highly feasible the interdomain motions of these proteins are coupled to binding events and are linked to signal transduction. On that note, the flexible stalks of the TCR, MHC, CD8, and CD3 molecules also likely play a role.

## Conclusions and Future Perspectives

Protein flexibility is inherent to protein structure and function, and TCR-pMHC systems are no exception. Despite this the systematic analysis of the flexibility of TCR-pMHC systems is lagging far behind that of other fields (103–105), particularly when it comes to integration of computational and experimental techniques.

We propose that to advance our mechanistic understanding of how TCR-pMHC engagement initiates intracellular signaling, and the influence of the peptide on the signal, that there needs to be a shift in our approach, both in terms of the suite of techniques used to assess flexibility, and use of creative engineering to surpass limitations specific to the molecules in question and their applicability to a technique. Elegant examples that illustrate the strength of this marriage are now emerging. For example, Natarajan et al. (38) overcame the size barrier that limits the study of the soluble TCR by NMR through the use of perdeuteration, and concomitantly simplified the NMR spectra using partial subunit labeling. This NMR approach combined with mutagenesis, computational docking, and validation using cell-based assays has enhanced our understanding of how the extracellular engagement of the TCR-CD3 complex transmits a signal. Likewise, Birnbaum et al. (106) implemented clever strategies to circumvent size limitations and issues pertaining to sample heterogeneity to use electron microscopy to observe the molecular architecture of the membrane-associated TCR-CD3 complex bound to pMHC. Using this approach combined with SAXS they put forward a ligand-dependent dimerization mechanism for TCR signaling in which flexibility plays a key role.

We also propose the ensemble refinement technique be used routinely in the X-ray crystallographic analysis of TCR-pMHC systems. The routine extraction of this data, and validation/interpretation in conjunction with other experimental techniques, some of which are summarized here, will provide previously hidden insights into the scope of conformational changes permissible by peptides when bound to MHC that influence TCR binding and T cell activation and will also reveal insights into how TCR flexibility and dynamically-driven allostery play a role. This hitherto missing information will enable us to more fully consider how a signal is transduced from the pMHC interface via the CD3 subunits and to determine how flexibility at the interface correlates with the degree of T cell stimulation (79, 107). This may provide new insights into how the T cell response can be therapeutically manipulated to fight infections or cancer. For example, by considering the flexibility of an MHC-bound peptide in conjunction with other peptide characteristics (such as amino acid sequence, prominence, solvent exposure, and affinity for MHC) we may more accurately predict epitope immunogenicity, particularly for neoantigen-based vaccine design (108–112). The use of polypeptide vaccines bearing HLA-restricted CD8+ T cell epitopes is fast gaining traction for cancer immunotherapy (108, 113, 114). The aim is to vaccinate individuals with mutated tumor-associated epitopes (mimotopes) that are then presented by MHC and in doing so stimulate CD8+ T cells that prevent tumor growth. Often mimotopes with enhanced binding to MHC and/or altered TCR interactions elicit a more effective tumor-specific T cell response (115–119) and so their rational design, facilitated by accurately predicting peptide immunogenicity (108, 120, 121), would be highly beneficial.

Likewise, the rational design, or engineering, of the antigen-binding site of TCRs with the same specificity, but enhanced affinity and kinetics for tumor antigens (which are mostly self-derived), has practical implications for soluble TCR-based therapy (, 113, 122) and adoptive T cell immunotherapies for cancer (123, 124). Both approaches require the considered engineering of high affinity TCRs that maintain their specificity for target tumor antigens. Although this has been accomplished using techniques such as directed evolution (125–130) and structure-based design (58) these experimental approaches are, in tandem, informing the development of computational approaches to predict how to manipulate TCR binding properties (58, 128), and there are indications that the accuracy of these computational approaches is enhanced by incorporating MD simulations for the consideration of protein flexibility (129, 131). However, application of simulation techniques lags markedly behind other fields, so conceptual advances will require highly integrated experimental and computational approaches to fully understand, and exploit, the dynamics of the system.

## Author Contributions

AB and NB wrote the manuscript and NB produced Figures 1,2.

### Conflict of Interest Statement

The authors declare that the research was conducted in the absence of any commercial or financial relationships that could be construed as a potential conflict of interest.
